# EUS-FNA Diagnosis with Core Biopsy of Pancreatic Metastases from Primary Breast Cancer

**DOI:** 10.1155/2020/7136897

**Published:** 2020-09-16

**Authors:** Samuel Galante Romanini, Juan Pablo Román Serrano, Juliana Silveira Lima de Castro, Isabela Trindade Torres, Alex Ingold, André Lucchiari Borini, Luiz Augusto Sanches Zulske, Maria Bruna Feitosa Matias, Jéssica Said de Marchi, José Andrés Sanchez Pulla, José Celso Ardengh

**Affiliations:** ^1^Gastrointestinal Endoscopy, Department of Gastrointestinal Endoscopy, Hospital 9 de Julho, São Paulo, Brazil; ^2^Department of Radiology, Irmandade da Santa Casa de Misericórdia de São Paulo, São Paulo, Brazil; ^3^Hospital do Servidor Público Municipal, São Paulo, Brazil; ^4^Gastrointestinal Endoscopy Service, Department of Gastrointestinal Endoscopy, Hospital das Clinicas, University of São Paulo Riberão Preto, São Paulo, Brazil

## Abstract

**Background:**

The pancreas as a site of metastasis of other primary tumors is a rare event. Pancreatic metastases may occur years after the start of treatment of a neoplasm of another organ or may be the initial manifestation of an unidentified primary tumor. The most commonly reported primary sites for pancreatic metastases are the kidneys, lungs, breast, bowel, and skin (melanoma). *Case Summary*. The authors report a case of pancreatic metastasis derived from a primary breast cancer that underwent endoscopic ultrasound fine-needle aspiration (EUS-FNA) core biopsy to obtain tissue, which made it possible to perform an immunohistochemical study.

**Conclusion:**

We emphasize the importance of outpatient follow-up after the treatment of a neoplasia and the completion of control exams. In addition, we should always be aware of the finding of a secondary lesion in patients who have already been diagnosed with cancer, even if it is located in unusual organs, as in this case, where two metastases of a breast carcinoma to the pancreas were detected.

## 1. Introduction

The pancreas as a site of metastasis of other primary tumors is uncommon and may be difficult to differentiate from a neoplasm derived from the exocrine and/or endocrine pancreas itself, due to the overlap of its clinical presentation, imaging, and cytological features [1].

Pancreatic metastases may occur years after the start of treatment for a neoplasm from another site or may be the initial manifestation of an unidentified primary tumor. The most commonly reported primary sites for pancreatic metastases are the kidneys, lungs, breast, bowel, and skin (melanoma) [1]. Diagnosis before therapy should be the cornerstone of the decision to be made. Pancreatic tissue biopsies accurately detect this type of disease and thus establish the best form of treatment.

The authors report a case of pancreatic metastasis derived from a primary breast cancer that underwent needle puncture to obtain tissue, the result of which allowed the immunohistochemical study.

## 2. Case Presentation

**Figure 1:**
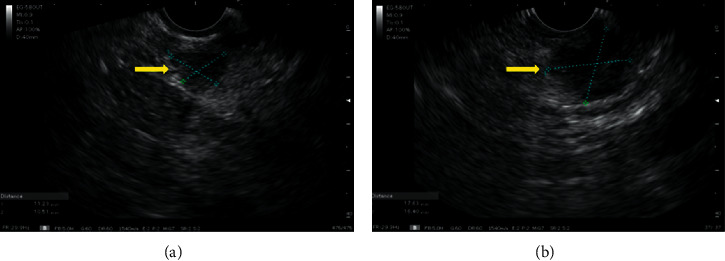
Sectorial scanning of uncinate process and tail of pancreas. (a) Hypoechoic lesion, homogeneous and well-delimited in the uncinate process and (b) another lesion with the same characteristics but larger in the pancreas tail.

**Figure 2:**
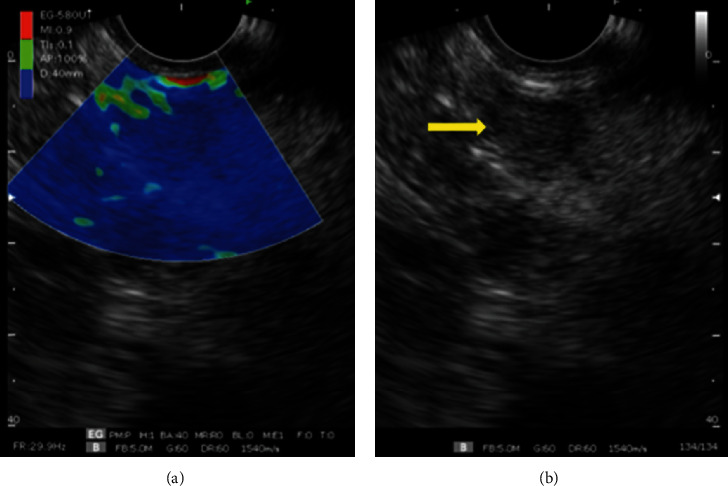
Elastography performed with a sectorial endoscopic ultrasound device. (a) Elastography of uncinate hardened process lesion (blue image). (b) Yellow arrow indicating hypoechoic lesion of uncinate process.

**Figure 3:**
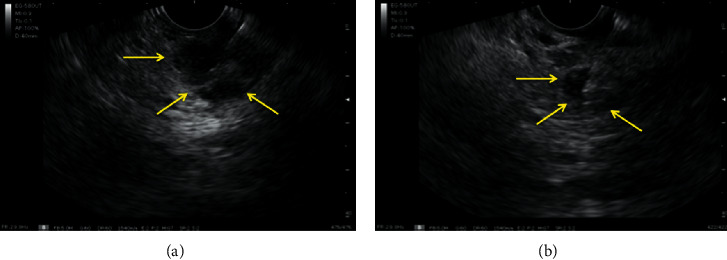
(a) and (b) EUS-FNA with Procore 20G needle.

A 72-year-old, asymptomatic, postmenopausal woman sought medical attention for routine exams with a personal history of systemic arterial hypertension, dyslipidemia, and previous appendectomy. She reported a right breast quadrantectomy in June 2016 by ductal breast adenocarcinoma (stage IIA) followed by radiotherapy. She had no family history of breast cancer and denied smoking or alcoholism. Her physical examination showed only a scar from previous surgery on her right breast. Abdominal ultrasonography on February 2019 detected a well-defined nodular lesion on the tail of the pancreas, approximately 2 cm in diameter and without compromising adjacent structures. Other imaging tests were not performed due to a significant allergy to radiological contrast. She underwent endoscopic ultrasound (EUS) which confirmed the finding of abdominal US but detected two more hypoechoic, homogeneous, round areas, with well-defined limits, of soft consistency to elastography, two located in the uncinate process and one in the pancreatic tail, measuring 7 × 8 mm, 11 × 10 mm, and 19 × 12 mm, respectively (Figures 1 and 2). They did not invade or compress the main pancreatic duct or vascular structures. EUS-FNA core biopsy was performed on all lesions with a ProCore 20G needle (Cook Medical) (Figure 3). An additional finding of the exam was the presence of gallbladder stones.

**Figure 4:**
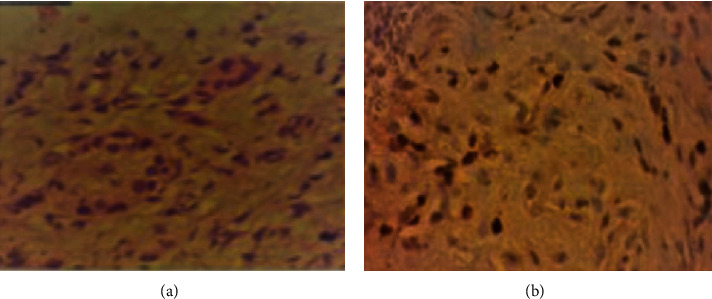
Material obtained through EUS-FNA and sent for histological and immunohistochemical study. (a) Hematoxylin-eosin revealing atypical microducts and (b) GATA3-labeled immunohistochemistry (+) -secondary breast carcinoma.

The histological results of the punctures revealed that the lesion located on the tail of the pancreas and the largest in the uncinate process were secondary microduct or small cell-associated epithelial neoplasia isolated (metastatic ductal carcinoma). The smallest located in the uncinate process was negative for neoplasia. The immunohistochemistry of the material was positive for the GATA3 marker, which concluded that it was an invasive carcinoma of mammary origin in the form of triple negative discesa cells (estrogen negative, progesterone negative, and HER2 negative receptors) (Figure 4). The patient was diagnosed with late metastases of breast cancer to the pancreas and resumed follow-up with the oncology team.

## 3. Final Diagnosis

Metastases of breast cancer to the pancreas.

## 4. Outcome and Follow-Up

The patient whose case was reported in this article resumed the follow-up with a specialized oncology team for definitive treatment of the diagnosed lesions.

## 5. Discussion

The pancreas as a site of metastasis is uncommon and difficult to differentiate from ductal adenocarcinoma due to its clinical presentation, imaging findings, and cytological features [1]. Pancreatic metastasis may occur years after the treatment of a primary neoplasm or may be identified even before its diagnosis, and the latter is much rarer than the first [1]. The most frequently identified primary sites for pancreatic metastasis are the kidneys, lungs, breast, bowel, and skin [1].

Few studies in the literature have evaluated the efficacy of EUS-FNA in the diagnosis of this type of lesion [2]. In a study by Fritscher-Ravens A et al. of 207 patients with focal pancreatic mass who underwent EUS-FNA, 116 were diagnosed with malignant neoplasia, of which 32 (27%) were nonmalignant primary pancreatic adenocarcinoma [3]. Recently, the use of harmonic contrast associated with endoscopic ultrasound (CH-EUS) has started to be applied as an adjuvant in the differential diagnosis of pancreatic neoplasms; this is due to the advantage of the method of being able to visualize both the parenchyma and the microvasculature without the artifacts caused by Doppler [4]. The finding of lesions with a pattern of hyperenhancement to the use of contrast, associated, in particular, with a history of hematological or renal neoplasia, are strong predictors for pancreatic metastatic lesion (lymphoma and renal carcinoma metastasis), in some selected cases avoiding, up to even, the EUS-FNA [4].

Pancreatic ductal adenocarcinomas (ADPs) are the most common solid tumors, while others such as neuroendocrine and metastases are the rarest forms [5]. Breast cancer accounts for less than 5% of metastatic pancreatic lesions [6]. Invasive lobular breast carcinoma is the type of breast cancer that most metastasizes to the digestive tract [6]. Ductal carcinoma typically exhibits solitary metastasis and is much rarer in a diffuse pattern in the digestive tract [6].

Immunohistochemistry of the material obtained by puncture is an important tool to confirm the origin of metastatic carcinoma. Mammoglobin (MGB), GCDFP-15, estrogen receptors (ER), and progesterone (PR) act as specific immunohistochemical markers in the evaluation of metastatic breast carcinoma [7]. GATA3 is a member of the zinc-finger transcription factor family [7]. This marker plays an important role in breast development and cell differentiation of the luminal epithelium as well as in the development of other tissues and has been implicated in breast cancer growth, differentiation, progression, and metastasis [7]. GATA3 is widely expressed in estrogen receptor negative luminal breast cancer and is more sensitive than GCDFP-15 and MGB, making it a useful marker for breast carcinoma [7].

The 5-year survival of invasive breast cancer is 90%, but when there are distant metastases, a typical ductal adenocarcinoma becomes incurable and a 5-year survival of approximately 25% [6]. Those with metastasis to the pancreas have a survival range of 1 to 50 months [6].

There is controversy regarding the resection of pancreatic metastases [6]. There are reports that resection of isolated pancreatic lesions may increase the survival and therefore should be the conduct of choice, while other authors advocate systemic treatment initially due to morbidity and mortality associated with duodenopancreatectomy [6].

Among chemotherapeutic drugs, taxanes, docetaxel, and paclitaxel are the drugs of choice in the treatment of metastatic breast cancer [8]. They can be used as single agents or in combination with other chemotherapeutic drugs [8].

## 6. Conclusion

In conclusion, we emphasize the importance of outpatient follow-up after the treatment of a neoplasm and the completion of control exams. In addition, we should always be aware of the finding of a secondary lesion in patients who have already been diagnosed with cancer, even if it is located in unusual organs, as in this case, where two metastases of a breast carcinoma to the pancreas were detected.

## Data Availability

The data used to support the article are available within the article.
